# Geographical Variation in Skull Morphology of a Wild Rodent (*Orientallactaga sibirica*) Across Environmental Gradients

**DOI:** 10.1002/ece3.73236

**Published:** 2026-03-09

**Authors:** Cheng Yang, Rui Geng, Haizhou Yang, Yongling Jin, Zhenghaoni Shang, Yakun Liu, Xiaodong Wu, Heping Fu, Shuai Yuan

**Affiliations:** ^1^ College of Grassland Science Inner Mongolia Agricultural University Hohhot China; ^2^ Key Laboratory of Grassland Resources of the Ministry of Education Hohhot China; ^3^ Key Laboratory of Grassland Rodent Ecology and Pest Controlled Hohhot China

**Keywords:** cranial adaptation, geographical variation, geometric Morphometrics, *Orientallactaga sibirica*, skull morphology

## Abstract

*Orientallactaga sibirica* ( *O. sibirica* ), a member of the family Dipodidae, is widely distributed across Central Asia and plays a significant role in grassland ecosystems. Although substantial ecological data exist for this species in China, research on intraspecific cranial variation is limited, and no comprehensive surveys have been conducted across its distribution range in China. This study aims to address this gap by collecting specimens of *O. sibirica* from various geographic regions in China, conducting geometric morphometric analyses of their skulls, and examining the influence of current climatic conditions on cranial morphology. Our results show that significant cranial variation in *O. sibirica* is observed in the nasal, parietal, and maxillary regions near the nasal end, as well as the zygomatic arch and preorbital bridge. These differences cause skulls from northeastern China to cluster distinctly from those from the Qinghai‐Tibet Plateau. Regression analyses indicated that skull size is primarily associated with annual precipitation, whereas skull shape is significantly associated with altitude. Our findings reveal a distinct morphological pattern in the Qinghai–Tibet Plateau population, suggesting a high degree of geographic differentiation that warrants further investigation. Characterizing environment‐associated intraspecific variation provides a baseline for understanding morphological diversity in *O. sibirica* across China.

## Graphical abstract

The greatest morphological differences are observed between the northeastern regions of China and the Qinghai‐Tibet regions. Elevation and temperature are the primary climatic factors causing morphological variation. Compared to the current period, the future suitable habitat area for Five‐toed jerboa is expected to decrease and shift toward higher latitudes.

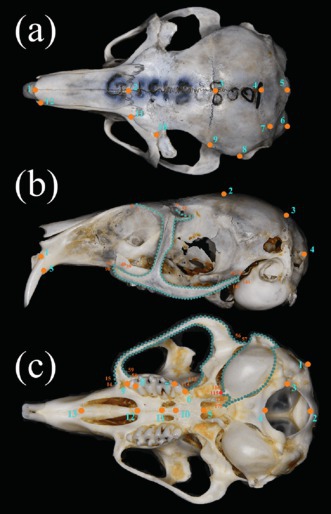

## 1 Introduction

The *Orientallactaga sibirica* ( *O. sibirica* ), belonging to the subfamily Allactaginae, is a nocturnal, hibernating rodent well‐adapted to open terrain. Key morphological traits include long hind feet for rapid locomotion, shorter forelimbs for feeding and digging, and enlarged auditory bullae for predator detection. Its tail, approximately 1.5 times its body length, provides support when standing upright (Alhajeri and Steppan 2018; Lebedev et al. 2013; Michaux et al. 2017). Widely distributed across the steppes and deserts of Central Asia, *O. sibirica* occupies a vast range in northern China, from Jilin to Xinjiang, demonstrating remarkable adaptability to diverse environmental conditions.

The skull is a complex structure whose morphology is shaped by a combination of genetic, developmental, and environmental factors. At the intraspecific level, cranial variation can provide deep insights into a population's genetic structure, ecological adaptation, and evolutionary trajectory (Fernandes et al. 2009; Lebedev et al. 2013). Indeed, environmental factors are recognized as major drivers of cranial variation in rodents (Alhajeri 2018; Martínez and Di Cola 2011; Monteiro et al. 2003; Quiroga‐Carmona et al. 2023). Recent work documented environmentally associated phenotypic variation in body size and trophic niche across changing habitats (Buren et al. 2025). Together, these findings motivate a complementary focus on cranial morphology as an additional axis of phenotypic variation potentially shaped by environmental gradients. Although significant cranial variation has been well‐documented at the subfamily level (Alhajeri 2021), and cranial morphometrics has proven effective for distinguishing even closely related rodent species (e.g., Tabatabaei Yazdi and Adriaens 2013), a comprehensive analysis of how skull morphology varies within *O. sibirica* across China's distinct environmental landscapes remains a critical knowledge gap.

To precisely quantify this complex morphological variation, we used geometric morphometrics (GMM). As a powerful analytical framework, GMM surpasses traditional linear measurements in its ability to capture and visualize subtle shape differences in biological structures (Bookstein 1997; Richtsmeier et al. 2002; Rohlf 2015). This technique is now widely applied in studies of rodent cranial morphology (D'Anatro and Lessa 2006; Demirbaş et al. 2023; Kang et al. 2023), allowing for robust comparisons across populations and revealing patterns of adaptation to local conditions (Adams et al. 2004; De and Dwivedi 2023; Viacava et al. 2023).

This study aims to fill the aforementioned gap by conducting the first comprehensive survey of *O. sibirica* skull morphology across its environmental gradients in China. By correlating cranial morphology with key environmental factors, we aim to understand the adaptive strategies driving these differences. Specifically, we test the following hypotheses:

1. *Geographical structuring*: Skull morphology of *O. sibirica* is not uniform but exhibits significant geographical variation, with populations from distinct ecoregions forming discrete morphological clusters.
2. *Ecomorphological association*: Both skull size and shape are expected to correlate with key environmental variables. We predict that size variation may relate to thermoregulatory principles or resource availability, whereas shape will reflect adaptations to regional differences in climate, such as temperature and precipitation.
3. *Functional module variation*: Morphological variation will be concentrated in specific functional modules of the skull, such as the rostrum and zygomatic complex, reflecting adaptations to local selective pressures.

## 2 Materials and Methods

### 2.1 Skull Analysis

#### 2.1.1 Sample Collection

**FIGURE 1:**
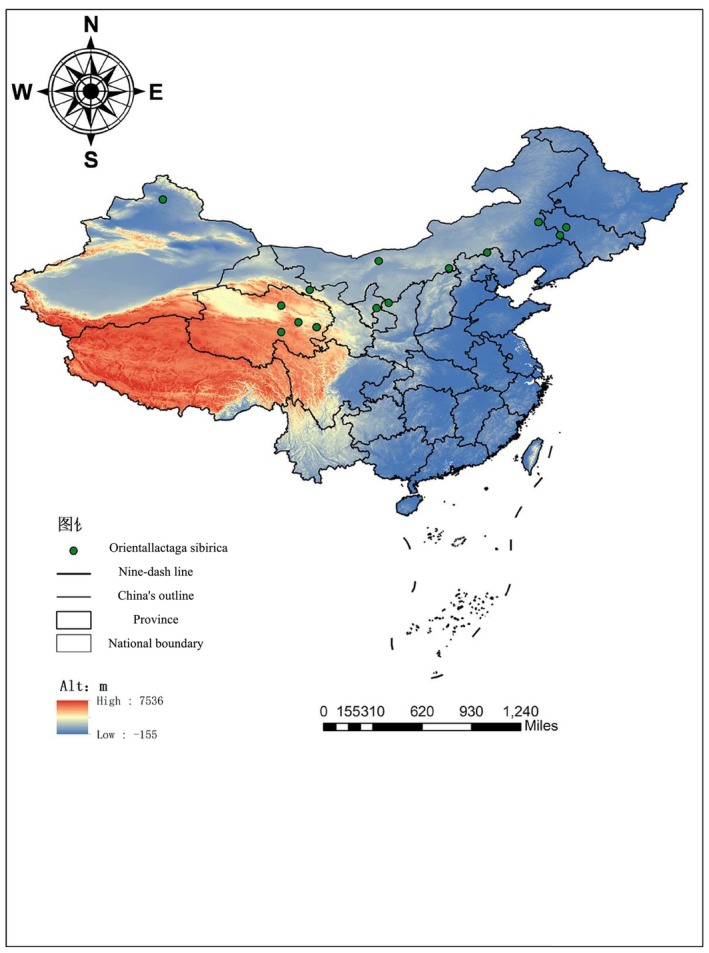
A map showing the sampling sites for Orientallactaga sibirica specimens in northern China.

A total of 138 adult specimens of *O. sibirica* (71 males, 67 females) were collected from 14 locations across the species' distribution range in China (Figure 1; Table 1). All specimens were obtained from wild populations between 2018 and 2020 using live traps. To ensure maximum consistency in preservation and data quality, and to avoid potential biases arising from varied preservation methods or ages in historical collections, this study focused exclusively on these recently collected specimens. The skulls were prepared for analysis by carefully removing soft tissue through gentle boiling and were subsequently whitened with hydrogen peroxide.

**TABLE 1:** Sampling information for the 14 geographical regions of Orientallactaga sibirica includes sample size and coordinates (latitude and longitude).

G10	Huan County, Gansu Province	6	106.8397	37.1050
G6	Minle County, Gansu Province	8	100.7489	38.7539
H6	Weichang County, Hebei Province	9	116.9025	42.1464
JL1	Shuangliao City, Jilin Province	11	123.5406	43.6967
JL3	Nong'an County, Jilin Province	13	124.1144	44.4475
M19	Urad Rear Banner, Inner Mongolia	9	107.0453	41.3794
M23	Fengzhen City, Inner Mongolia	11	113.4306	40.7075
M36	Xing'an League, Inner Mongolia	12	121.5808	44.9147
Q10	Maduo County, Qinghai Province	10	98.1718	34.9094
Q2	Guide County, Qinghai Province	10	101.3603	35.3439
Q4	Xinghai County, Qinghai Province	7	99.7175	35.7981
Q9	Tianjun County, Qinghai Province	9	98.1717	37.3103
S4	Dingbian County, Shaanxi Province	12	107.9411	37.5700
W5	Fuhai County, Xinjiang	9	87.3697	46.9667

#### 2.1.2 Landmarks

Following the methods of Abiadh et al. (2010), Alhajeri (2018), Colangelo et al. (2010), and Fornel et al. (2010), a Nikon D7500 digital SLR camera was mounted on a tripod with the lens positioned perpendicular to the ground. The camera was placed approximately 40 cm from the specimens to capture three views of the skulls (dorsal, ventral, and lateral) at a resolution of 4176 × 2784 pixels, with a flash used for additional lighting. Of the 138 specimens, 26 ventral and 37 lateral samples were excluded because of damage. Each photograph included a scale bar next to the skull to facilitate scaling and calculation of the centroid size (CS).

**FIGURE 2:**
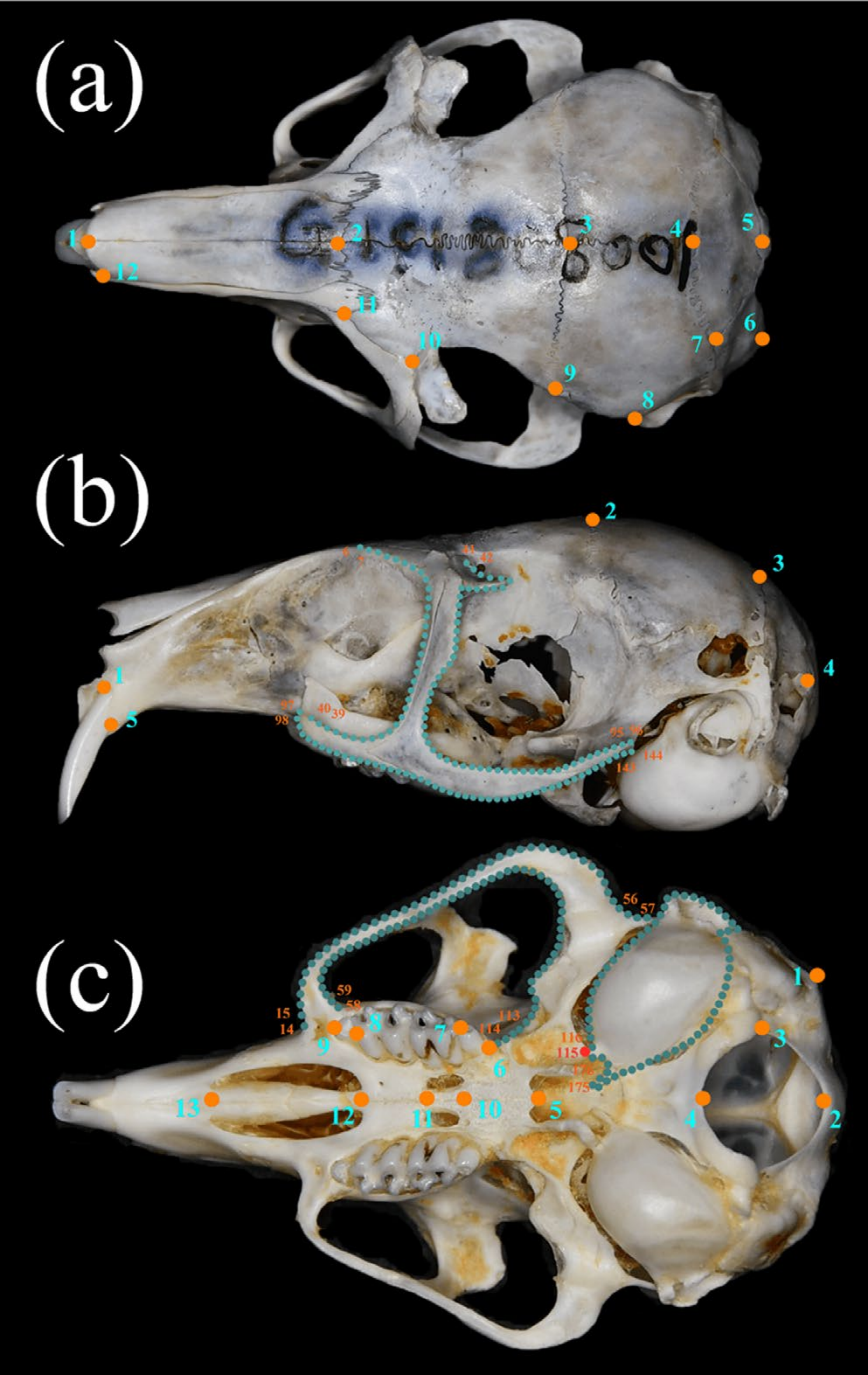
(a) Dorsal, (b) ventral, and (c) lateral views of the skull, showing the positions of landmarks (orange points) and semi‐landmarks (navy points). Red points indicate the starting position of the auditory bulla semi‐landmarks. Detailed descriptions are provided in Table 2.

The photos were converted to TPS format using *tpsUtil32* and digitized in *tpsDig 2.32* (Rohlf 2015). Fourteen landmarks were digitized on the dorsal view of each skull, 13 landmarks and 173 semi‐landmarks on the ventral view, and five landmarks and 139 semi‐landmarks on the lateral view (Figure 2, Table 2). These landmarks included both commonly used cranial landmarks and specific landmarks characteristic of *O. sibirica* (D'Anatro and Lessa 2006; Alhajeri 2021; Quintela et al. 2016). Each sample was scaled and numbered after digitization for subsequent software analysis.

**TABLE 2:** Definition of landmarks (semi‐landmarks) of Orientallactaga sibirica.

Landmarks (Semi‐landmarks)	Definition
Dorsal	
1	Anterior extremity of suture between nasals
2	Suture between the nasals and frontals
3	Suture between frontal and parietal
4	Suture between parietals and interparietal
5	Posterior‐most point of suture between the interparietal and occipital
6	Caudolateral end of the occipital bone in the dorsal projection
7	Intersection of the parietal‐interparietal and interparietal‐occipital sutures
8	Suture between the parietal and tympanic bulla in the dorsal projection
9	Suture between the frontals and parietal
10	Posteriormost point of suture between the antorbital bridge (maxillary), lachrymal, and frontal
11	Suture between the nasal, frontal, and premaxillary
12	Anteriormost point of suture between the nasal and premaxilla
Lateral	
1	Posteriormost point of the incisor alveolus
2	Superiormost point of suture between the frontal and parietal
3	Superiormost point of suture between the parietal and interparietal
4	Posteriormost point of occipital
5	Inferiormost point of the incisor alveolus
6–41	Semi‐landmarks along the antorbital bridge of the maxillary
42–96	Semi‐landmarks along the lachrymal and postorbital bridge
97–144	Semi‐landmarks along the inferorbital margin of zygomatic arch
Ventral	
1	Posteriormost point of occipital condyle
2	Posteriormost point of foramen magnum
3	Posteriormost point on the inside edge of the occipital condyle
4	Anteriormost point of the foramen magnum
5	Anteriormost point of the palatine bone
6	Posteriormost margin of the third molar
7	Anteriormost margin of the third molar
8	Anteriormost margin of the first premolar
9	Posteriormost margin of the first premolar
10	Mid‐point of the anteriormost point between the palatine foramen
11	Mid‐point of the anteriormost point between the palatine foramen
12	Mid‐point of the anteriormost point between the incisive foramen
13	Mid‐point of the posteriormost point between the incisive foramen
14–57	Semi‐landmarks along the margin of the zygomatic arch
58–114	Semi‐landmarks along the margin of the zygomatic arch
115–176	Semi‐landmarks along the tympanic bulla

### 2.2 Geometric Morphometric and Statistical Analyses

#### 2.2.1 Procrustes Superimposition and Shape Variables

Raw landmark coordinates were imported into *MorphoJ 1.08* (Klingenberg 2011). To separate size and shape information, a Generalized Procrustes Analysis (GPA) was performed. GPA standardizes the landmark configurations of all specimens by translating them to a common centroid, rotating them to a minimal squared distance, and scaling them to a unit CS. This process removes all non‐shape variation, yielding two variables: CS, an isometric estimator of overall skull size, and a set of Procrustes coordinates, which represent the pure shape of each skull.

#### 2.2.2 Analysis of Morphological Variation

We tested for significant differences in skull shape among the 14 geographical groups using a Procrustes analysis of variance (ANOVA). To visualize the major patterns of shape variation, we conducted a principal component analysis (PCA) to explore the main axes of variance in the entire dataset, and a canonical variate analysis (CVA) to maximize the separation among the pre‐defined geographical groups. The significance of group separation in the CVA was assessed with a permutation test (10,000 rounds). We also performed a UPGMA clustering analysis based on Procrustes distances using the “ape” package in R (Paradis and Schliep 2019) to visualize the phenetic relationships among populations.

### 2.3 Ecomorphological Analyses

#### 2.3.1 Environmental Data

Nineteen bioclimatic variables and elevation data were sourced from the WorldClim 2.1 database at a 2.5 arc‐minute resolution (Fick and Hijmans 2017; Table 3). Crucially, we utilized the standard 1970–2000 climatic data, as this period represents a long‐term climatic baseline. This baseline captures broad, persistent environmental gradients across the study area and helps reduce the influence of short‐term climatic fluctuations around the sampling period (2018–2020).

**TABLE 3:** Environmental variables used in Regression analysis.

Variables	Definition
Bio1 (°C)	Annual Mean Temperature
Bio2 (°C)	Mean Diurnal Range (Mean of monthly (max temp–min temp))
Bio3	Isothermality (BIO2/BIO7) (×100)
Bio4	Temperature Seasonality (standard deviation ×100)
Bio5 (°C)	Max Temperature of Warmest Month
Bio6 (°C)	Min Temperature of Coldest Month
Bio7 (°C)	Temperature Annual Range (BIO5‐BIO6)
Bio8 (°C)	Mean Temperature of Wettest Quarter
Bio9 (°C)	Mean Temperature of Driest Quarter
Bio10 (°C)	Mean Temperature of Warmest Quarter
Bio11 (°C)	Mean Temperature of Coldest Quarter
Bio12 (mm)	Annual Precipitation
Bio13 (mm)	Precipitation of Wettest Month
Bio14 (mm)	Precipitation of Driest Month
Bio15	Precipitation Seasonality (Coefficient of Variation)
Bio16 (mm)	Precipitation of Wettest Quarter
Bio17 (mm)	Precipitation of Driest Quarter
Bio18 (mm)	Precipitation of Warmest Quarter
Bio19 (mm)	Precipitation of Coldest Quarter
Attitude	

#### 2.3.2 Linking Morphology to Environment

Because cranial morphology may reflect both genetically based differentiation and non‐heritable phenotypic plasticity, our analyses are framed as tests of statistical associations between morphology and environmental variables rather than direct evidence of local adaptation. In particular, the use of broad‐scale climatic and elevational predictors allows us to evaluate whether geographic patterns in skull size and shape covary with persistent environmental gradients, which could arise through either evolutionary divergence or plastic responses.

To investigate the association between environmental factors and cranial morphology, we employed Procrustes linear regression analyses using the procD.lm function in the geomorph R package (Adams et al. 2013). We constructed two separate linear models to test our hypotheses:

1. *For skull size*: We modeled CS as a function of the selected environmental variables.
2. *For skull shape*: We modeled the multivariate Procrustes shape coordinates as a function of the same environmental variables.

For both models, statistical significance was assessed using a residual randomization permutation procedure (RRPP) with 999 iterations. To visualize the results, we identified the environmental variable with the highest explanatory power (highest R^2^) from the Procrustes ANOVA results. We then performed a PCA on the shape data using gm.prcomp and plotted the first principal component (PC1) scores and CS against these key environmental drivers to illustrate the evolutionary trends.

## 3 Results

### 3.1 Variation in Skull Size

**FIGURE 3:**
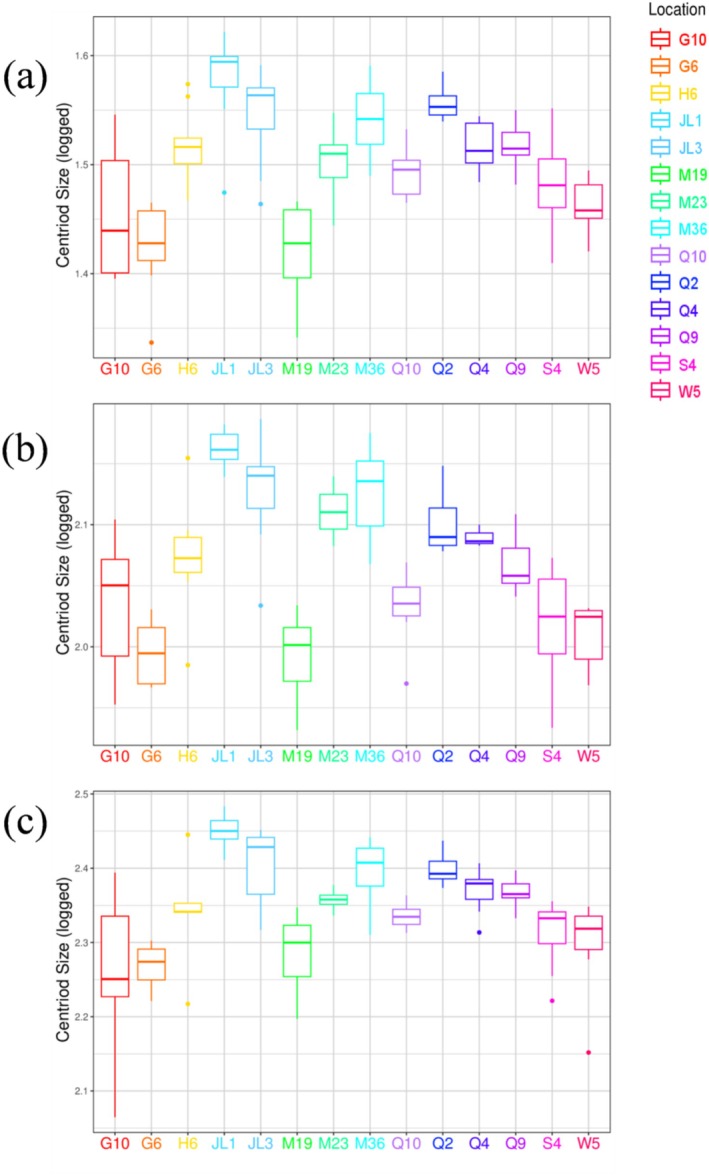
Box plots of the logged cranial centroid size for the dorsal (a), lateral (b), and ventral (c) views of the 14 geographic groups of *O. sibirica* . Each box plot displays the median (internal line), the 25th and 75th percentiles (box edges), the range (whiskers), and outliers (dots).

We first assessed the variation in overall skull size. An analysis of CSs revealed significant differences among the 14 geographical populations (*p* < 0.0001, Table 4). The boxplots show that populations from the Qinghai‐Tibet Plateau (QTP) (Q2, Q4, Q9, and Q10) consistently exhibited the largest skull sizes on average (Figure 3).

**TABLE 4:** The ANOVA analysis of centroid size and Procrustes coordinates across 14 geographical regions indicates significant correlations, as highlighted in bold.

	Sample size	SS	MS	df	*F*	*p*
(A) Centriod size						
Dorsal	138	5.9388	0.4568	13	19.17	**< 0.0001**
Ventral	112	38.8205	2.9862	13	13.00	**< 0.0001**
Lateral	101	20.0839	1.5449	13	19.25	**< 0.0001**
(B) Procrusters shape						
Dorsal	138	0.0564	0.0002	260	6.19	**< 0.0001**
Ventral	112	0.1024	2.2632E‐05	4524	4.12	**< 0.0001**
Lateral	101	0.1080	2.9243E‐05	3692	3.06	**< 0.0001**

### 3.2 Overall Patterns of Skull Shape Variation (PCA)

**FIGURE 4:**
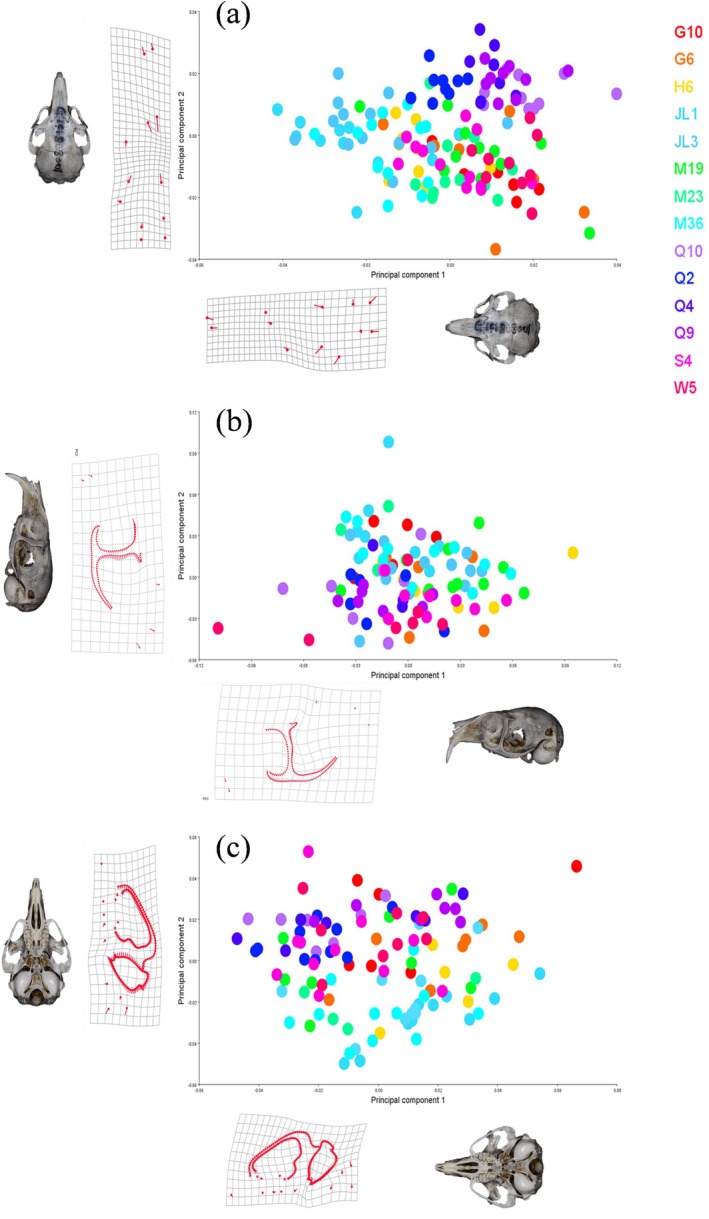
PCA of the geometric morphology for the dorsal (a), lateral (b), and ventral (c) views, using PC1 and PC2 as the *x* and *y* axes, respectively. The deformation grids illustrate the shape changes along each axis, with sample visualizations providing reference points for the direction of the grid deformations.

To explore the major axes of skull shape variation, we conducted a Principal Component Analysis (PCA). For the dorsal view, the first two principal components (PC1 and PC2) accounted for a combined 61.98% of the total variance. Although there was considerable overlap among most populations, the PCA plot revealed a clear separation of the four QTP populations from all other groups, primarily along the PC2 axis (Figure 4a). The shape changes associated with this axis indicated that QTP skulls are characterized by an expansion of the nasal bone and a contraction of the frontal bone. This initial exploratory analysis provided the first evidence for the unique morphology of the QTP populations.

For the lateral and ventral views (Figure 4b,c), the PCA did not produce such a clear separation. The populations showed extensive overlap, suggesting that the most dominant axes of overall shape variation within the entire dataset are not strongly aligned with the geographical groupings in these views. Nevertheless, the shape changes associated with the principal components were biologically informative, primarily relating to variation in the height of the zygomatic arch and the shape of the rostrum (lateral view), and the width of the palate and size of the incisive foramen (ventral view). This indicates that although geographical structure is less obvious in the overall variance, significant shape changes are still present in these key functional areas.

### 3.3 Maximizing Among‐Group Differentiation (CVA)

**FIGURE 5:**
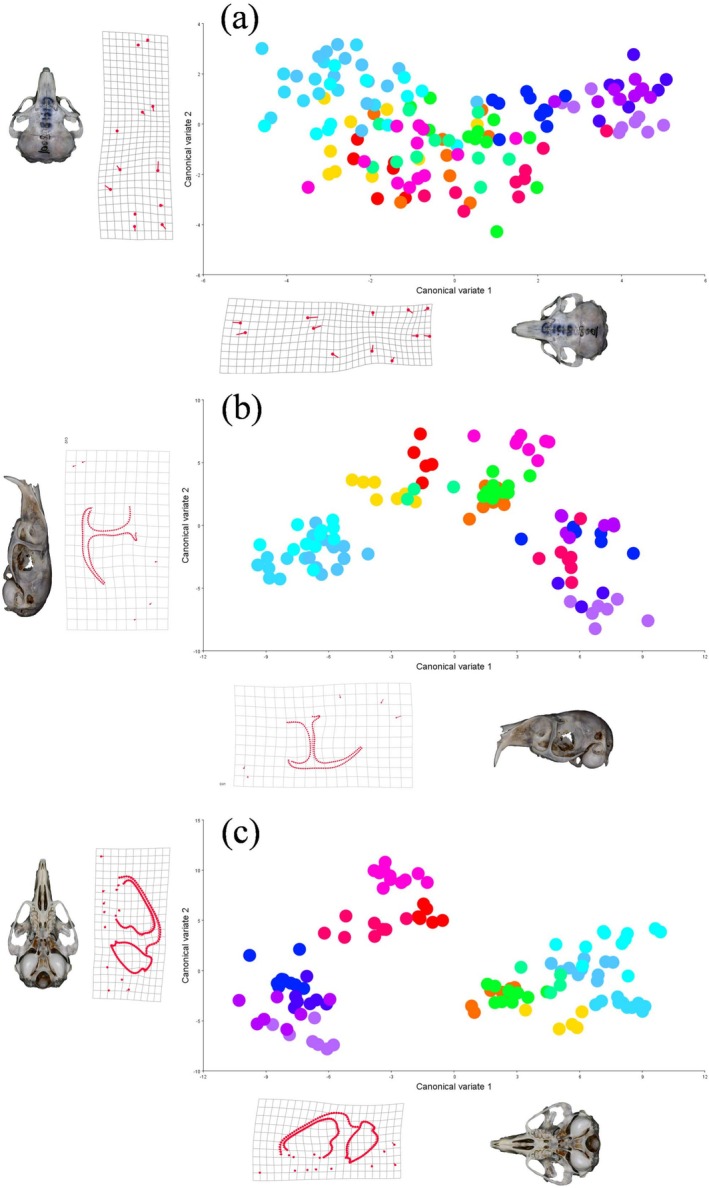
Canonical Variate Analysis (CVA) of cranial shape for the dorsal, lateral, and ventral views, plotted along the first two canonical axes (CV1 and CV2). Deformation grids illustrate the morphological variation associated with each axis, with accompanying skull photographs providing anatomical reference for the direction of the grid deformations.

Because CVA is designed to maximize differences among predefined groups, we interpret the CVA patterns as a confirmatory description of among‐region differentiation, complementary to the PCA results. The CVA results for the dorsal view of the skull (Figure 5a) reveal significant morphological differences along the CV1 axis (50.43%). The regions JL1, JL3, and M36 are clearly distinguished from regions Q2, Q4, Q9, and Q10, with the primary morphological features associated with this separation being the anterior–posterior expansion of the nasal bone, inward contraction of the frontal bone, and inward contraction of the parietal bone. These features contribute to a distinct cranial shape in regions from northeastern China (JL1, JL3, and M36) compared to those from the Qinghai‐Tibet Plateau. The CV2 axis (14.59%) did not show effective clustering, as regions exhibited considerable overlap, suggesting that other factors (such as sample size or environmental influences) may also contribute to the variation.

In the lateral view (Figure 5b), JL1, JL3, and M36 show a strong clustering pattern, whereas M23 clusters closely with G6, and Q2, Q4, Q9, and Q10 group together. The greatest distance is observed between JL1, JL3, and M36, indicating clear shape differences. The morphological changes driving this separation include constriction between the maxillary bridge and the zygomatic arch, as well as the outward displacement of the parietal and interparietal bones. Interestingly, Q9 and Q10 are close along the CV1 axis (34.85%) but show significant differences along the CV2 axis (18.85%). The morphological features associated with CV2 include the dorsal displacement of the incisor alveolar bone and convergence of the upper and lower ends of the maxillary bridge.

For the ventral view (Figure 5c), significant morphological variation is observed across regions, especially along the CV1 axis (38.77%). The four Qinghai regions (Q2, Q4, Q9, and Q10) are clearly distinguished from other regions, although there is significant sample overlap, particularly among Q4 and Q9, G10 and W5, and JL3 and M36. The morphological changes associated with these differences include the inward contraction of the auditory bullae and the outward expansion of the zygomatic arch near the nasal bone. Along the CV2 axis (16.87%), differences between Q2 and Q10, M23 and M36, and S4 and W5 are driven by the outward expansion of the posterior end of the zygomatic arch (squamosal) and a lateral deviation of the incisive foramen. Overall, the clear and predictable separation of populations from distinct ecoregions (e.g., QTP or northeastern plains) across all three views supports Hypothesis 1, whereas acknowledging the overlap observed in the PCA, especially for lateral and ventral views.

### 3.4 Phenetic Relationships Among Populations (UPGMA)

**FIGURE 6:**
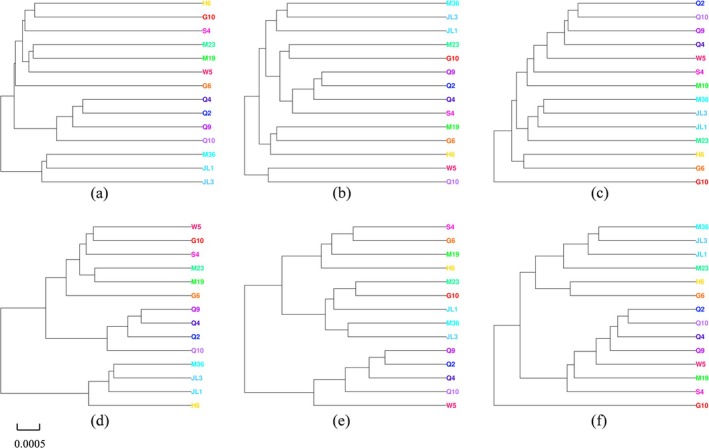
The Unweighted Pair Group Method with Arithmetic Mean (UPGMA) trees based on Euclidean distance for the dorsal (a), lateral (b), and ventral (c) views, and based on shape distance for the dorsal (d), lateral (e), and ventral (f) views. The scale is indicated at the bottom.

The UPGMA clustering analysis (Figure 6) offers valuable insights into the relationships among different geographic regions on the basis of cranial morphology. The clustering patterns for the dorsal view of the skull (Figure 6a,d) are consistent, both when using Euclidean distance (Figure 6a) and shape distance (Figure 6d). The regions JL1, JL3, M36, and H6 consistently cluster together, suggesting that these regions share similar cranial morphological characteristics. The four regions in the Qinghai‐Tibet Plateau (Q2, Q4, Q9, and Q10) form a separate, distinct cluster, indicating that these regions exhibit clear morphological differences from the northeastern groups. Additionally, the two Gansu regions (G6, G10) also form a separate cluster, reflecting their unique morphological traits compared to other regions.

For the ventral view (Figure 6b,e), the clustering patterns remain similar. The four Qinghai regions (Q2, Q4, Q9, and Q10) continue to form a tight cluster, whereas JL1, JL3, and M36 show close proximity, indicating that these regions are morphologically similar. The Gansu regions (G6 and G10) are consistently grouped together in both Euclidean and shape distances, supporting the findings from the dorsal view analysis.

In the lateral view (Figure 6c,f), the clustering results remain consistent with the other views. The Qinghai regions are closely grouped, and the northeastern regions (JL1, JL3, and M36) exhibit a distinct grouping. This clustering pattern highlights consistent geographic structuring in cranial morphology across China, suggesting that populations from the Qinghai–Tibet Plateau and northeastern China are phenetically distinct. These robust clustering patterns (Figure 6) are consistent with Hypothesis 1, which predicted significant and predictable geographical structuring in the skull morphology of *O. sibirica* . Furthermore, the observation that the differentiation is driven by changes in specific cranial regions, such as the nasal bone, zygomatic arch, and auditory bullae, as identified in the CVA, lends direct support to Hypothesis 3.

### 3.5 Association of Skull Shape With Environmental Factors

**FIGURE 7:**
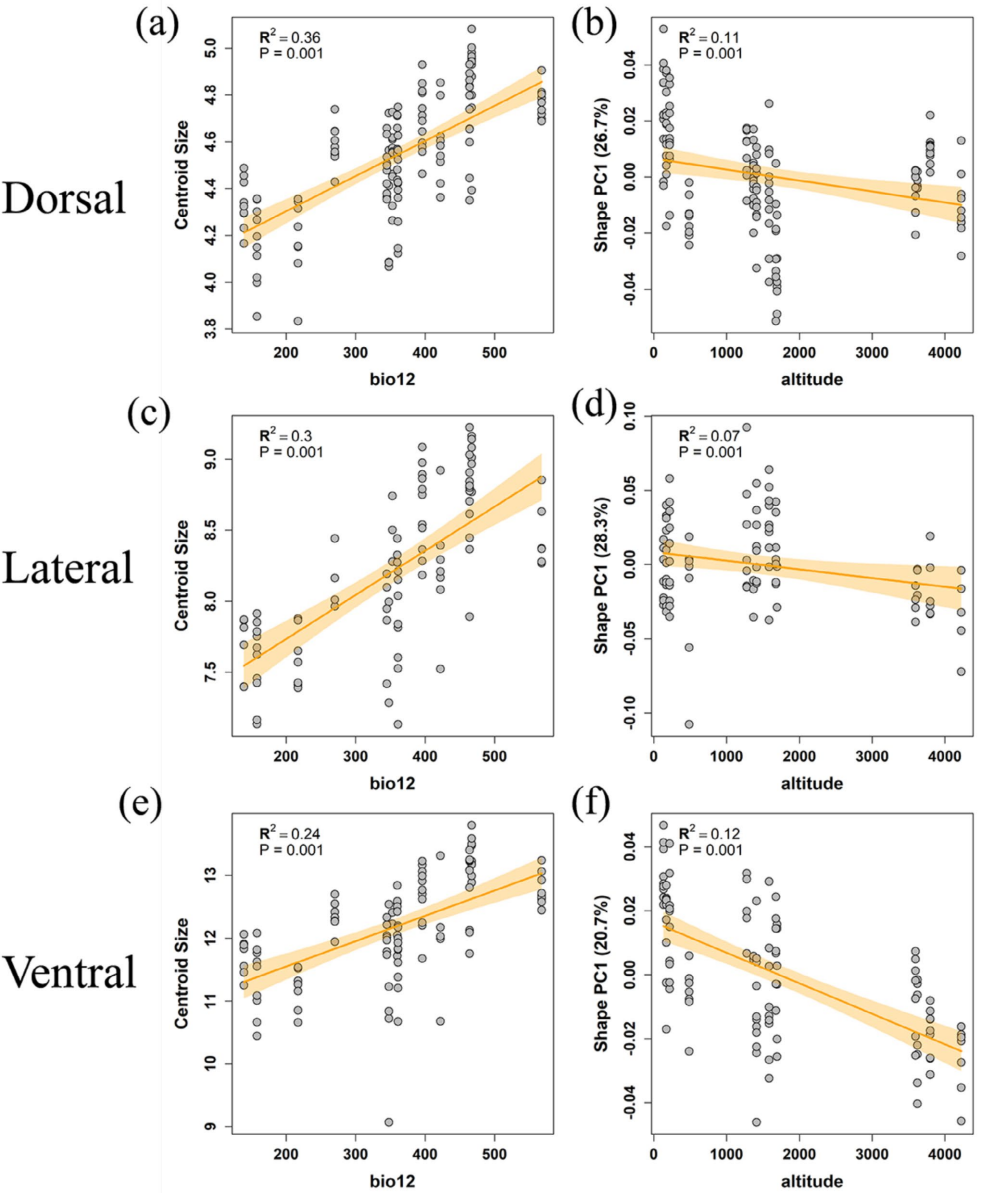
Regression analyses of skull morphology and environmental variables. (a, c, e) Relationships between centroid size and BIO12 for the dorsal, lateral, and ventral surfaces of the skull, respectively. (b, d, f) Relationships between shape PC1 and altitude for the dorsal, lateral, and ventral surfaces, respectively. Shaded areas indicate 95% confidence intervals.

The Procrustes ANOVA results for CS revealed that Annual Precipitation (bio12) was the most significant predictor across all views (*p* = 0.001). The regression models indicated that precipitation explains a substantial portion of the size variation (Dorsal: *R* ^2^ = 0.36; Lateral: *R* ^2^ = 0.30; Ventral: *R* ^2^ = 0.24, Figure 7, and Table 5). As visualized in the regression plots, there is a clear positive linear relationship, indicating a positive association between skull size and precipitation. The Procrustes linear regression analyses for shape indicated that altitude was the strongest environmental predictor of cranial morphology among the tested variables. The regression of shape variables against altitude was statistically significant for all three views (*p* = 0.001, on the basis of 999 permutations).

**TABLE 5:** Results of the regression analysis of cranial centroid size on environmental factors; bold indicates statistical significance.

Factor	SS	MS	Rsq	*p*
Dorsal				
Altitude	0.0800	0.07999	0.00904	0.108
Bio2	0.0722	0.07216	0.00816	0.142
Bio9	1.5305	1.53049	0.17300	**0.001**
Bio12	3.1437	3.14371	0.35535	**0.001**
Lateral				
Altitude	2.5087	2.5087	0.08997	**0.001**
Bio2	0.0455	0.0455	0.00163	0.524
Bio9	6.6702	6.6702	0.23920	**0.001**
Bio12	8.2772	8.2772	0.29683	**0.001**
Ventral				
Altitude	1.087	1.0865	0.01469	**0.073**
Bio2	1.749	1.7494	0.02365	**0.028**
Bio9	16.245	16.2447	0.21963	**0.001**
Bio12	17.536	17.5362	0.23709	**0.001**

To visualize this trend, we plotted the first principal component (PC1) of shape variation obtained via gm.prcomp against altitude (Figure 7). The plots reveal a significant negative relationship, demonstrating that individuals from high‐altitude regions (QTP) possess distinct cranial features compared to those from lower elevations. Although the total variance explained by altitude is moderate (Dorsal: *R* ^2^ = 0.11; Lateral: *R* ^2^ = 0.07; Ventral: *R* ^2^ = 0.12, Figure 7 and Table 6), the high statistical significance indicates that cranial shape covaries consistently with altitude across all views, thereby shaping the unique cranial architecture of *O. sibirica*. Detailed statistical outputs are provided in Tables 5 and 6.

**TABLE 6:** Results of the regression analysis of cranial shape on environmental factors; bold indicates statistical significance.

Factor	SS	MS	Rsq	*p*
Dorsal				
Altitude	0.020400	0.0203996	0.10867	**0.001**
Bio2	0.004883	0.0048830	0.02601	**0.002**
Bio9	0.013065	0.0130649	0.06960	**0.001**
Bio12	0.010631	0.0106314	0.05663	**0.001**
Lateral				
Altitude	0.021959	0.0219588	0.07028	**0.001**
Bio2	0.006202	0.0062023	0.01985	**0.046**
Bio9	0.007095	0.0070948	0.02271	**0.025**
Bio12	0.004933	0.0049329	0.01579	0.141
Ventral				
Altitude	0.028159	0.0281587	0.11894	**0.001**
Bio2	0.004340	0.0043403	0.01833	**0.007**
Bio9	0.013954	0.0139543	0.05894	**0.001**
Bio12	0.009520	0.0095198	0.04021	**0.001**

## 4 Discussion

This study, the first comprehensive geometric morphometric analysis of *O. sibirica* in China, clearly indicates that its cranial morphology is not uniform but is highly structured geographically. Our central finding is a marked morphological divergence between populations from the northeastern meadow steppes and those from the high‐altitude alpine steppes of the Qinghai‐Tibet Plateau (QTP). This result is consistent with our primary hypothesis and aligns with broader patterns in the Allactaginae subfamily, where cranial morphology is highly responsive to environmental gradients (Alhajeri 2021). Indeed, the existence of substantial intraspecific geographical variation in skull morphology is a well‐documented phenomenon across a wide range of rodent taxa (Dos Reis et al. 2002; Monteiro et al. 2003; Macholán et al. 2008; Damasceno and Astúa 2016; Quintela et al. 2016; Quiroga‐Carmona et al. 2023), confirming that our findings for *O. sibirica* are situated within a broad and consistent macroevolutionary pattern.

Our analyses reveal that skull size and shape are influenced by different environmental factors, suggesting complex adaptive pathways. Skull size was not primarily driven by temperature. Instead, our regression analysis demonstrated a strong positive relationship with annual precipitation. The fact that some of the largest individuals were found in the cold QTP region, whereas smaller individuals were found in the arid W5 basin, suggests that resource availability, which is tightly linked to precipitation, may be a more potent selective force on body size than temperature alone in these ecosystems (Monteiro et al. 2003). Importantly, Buren et al. (2025) also reported environmentally associated body‐size variation in *O. sibirica* across habitat gradients in China. Together, their findings and our precipitation–size relationship suggest that body size in this species covaries with broad‐scale environmental conditions, although the relative roles of genetic differentiation and phenotypic plasticity remain to be tested. Skull shape, in contrast, exhibited a significant linear relationship with altitude. The unique morphology of the QTP populations is likely associated with the extreme conditions of the plateau, as evidenced by the significant regression of shape variables against elevation. This association between cranial shape and local geoclimatic conditions is a recurring theme in arid‐adapted rodents. For instance, similar patterns of environmentally driven cranial divergence have been documented in jirds ( *Meriones persicus* and *M. crassus* ) from the geographically complex Iranian Plateau, providing a compelling parallel to our findings (Tabatabaei Yazdi and Adriaens 2011; Tabatabaei Yazdi et al. 2014).

The observed shape changes are concentrated in key functional modules, which is consistent with adaptive differentiation but does not exclude random genetic drift. The most notable feature is the elongation of the nasal bone in the QTP populations. An elongated rostrum houses more complex internal nasal turbinates, which act as a highly efficient “heat and moisture exchanger” (Noback et al. 2011). During inhalation in the cold, dry QTP environment, this large surface area warms and humidifies air before it reaches the lungs, preventing tissue damage and conserving water. This strongly suggests that the nasal morphology in QTP populations contributes to thermoregulation and respiratory efficiency under hypoxic and cold stress, but direct functional or physiological tests would be required to confirm this interpretation. Intriguingly, the QTP populations also exhibited relatively smaller auditory bullae. This finding appears to contradict the general rule that rodents in open habitats evolve larger bullae for enhanced predator detection (Alhajeri and Steppan 2018). This deviation suggests a more complex scenario on the QTP. Potential explanations include: (1) structural trade‐offs, where the cranial architecture required for respiratory adaptations constrains the size of the auditory bullae; or (2) a different acoustic environment on the high‐altitude steppe, where the selective advantage of large bullae is diminished.

Geographic isolation is a powerful engine of evolutionary divergence. The vast distances and formidable ecological barriers, particularly the QTP itself, may impede gene flow between the eastern and western populations. This restriction of gene flow allows each population to follow an independent evolutionary trajectory, where local adaptations (as discussed above) and genetic drift can lead to the fixation of different alleles and the accumulation of morphological differences (Wright 1943). A key question is whether these morphological differences reflect genetic adaptation or non‐heritable phenotypic plasticity. Although we cannot definitively rule out plasticity without common‐garden experiments, the magnitude of the differences and their clear functional relevance to the local environment (e.g., the nasal structure) are consistent with genetically based local adaptation; however, phenotypic plasticity remains a plausible alternative (or complementary) explanation that warrants explicit testing (e.g., common‐garden experiments, reciprocal transplants, or genomic analyses).

It is also crucial to consider the potential role of allometry (size‐related shape changes), as the QTP populations were also the largest. It is plausible that some of the observed shape variation is a simple byproduct of increased skull size. However, the specific and functionally relevant modifications in features like the rostrum and auditory bullae are unlikely to be mere consequences of being large. Future studies explicitly partitioning the allometric and non‐allometric components of shape will be critical to fully disentangle these effects.

Our findings have implications for the subspecies taxonomy of *O. sibirica* . Current classifications are debated, but our data reveal that the QTP populations are morphologically more distinct than any other recognized group. On the basis of this morphological evidence, we suggest that the *O. sibirica* populations on the Qinghai‐Tibet Plateau may represent a distinct evolutionary unit and could warrant further taxonomic assessment (e.g., at the subspecies level). This hypothesis requires rigorous testing. Future research should integrate genomic data (e.g., SNP or whole‐genome sequencing) with our morphological findings to evaluate the level of genetic divergence.

Furthermore, although our statistical results are robust, we acknowledge that future morphometric studies could benefit from even denser sampling, particularly in the vast central regions, to further refine the boundaries between these potential subspecies.

## 5 Conclusion

Through our study of *O. sibirica* across its distribution range in China, we found significant intraspecific geographic variation in skull size and shape, in contrast to previous research that often focused on interspecific variation. Notably, the morphological differences are most pronounced between the northeastern regions and the Qinghai‐Tibet Plateau, with shape changes primarily occurring in the nasal and parietal regions, the zygomatic arches (including the anterior maxillary region near the nasal end), and the preorbital bridge. Annual precipitation showed the strongest statistical association with skull size, whereas skull shape was most strongly associated with elevation among the tested variables. Our comparative analysis suggests that the skull morphology of *O. sibirica* on the Qinghai‐Tibet Plateau differs significantly from that of other regions. However, whether this population can be classified as a distinct subspecies requires further confirmation. Future genetic and common‐garden studies would be useful to determine whether these morphological differences are genetically based, reflect phenotypic plasticity, or involve both. Given the pronounced geographic differences in cranial morphology, our findings highlight the importance of considering local environmental conditions in species management strategies. Overall, our results indicate that morphological variation covaries with environmental gradients, providing a useful baseline for future studies and for conservation planning in regions with distinct environmental conditions.

## Author Contributions

**Cheng Yang:** software (equal), visualization (equal), writing – original draft (equal). **Rui Geng:** software (equal), visualization (equal), writing – original draft (equal). **Haizhou Yang:** software (equal). **Yongling Jin:** methodology (equal). **Zhenghaoni Shang:** investigation (equal). **Yakun Liu:** software (equal). **Xiaodong Wu:** methodology (equal). **Heping Fu:** funding acquisition (equal), project administration (equal), supervision (equal). **Shuai Yuan:** funding acquisition (equal), project administration (equal), supervision (equal), writing – review and editing (equal).

## Funding

This experiment was funded by the National Natural Science Foundation of China (32060256 and 32060395), Major Science and Technology Project of Inner Mongolia Autonomous Region (2021ZD0006), Science and Technology Project of Inner Mongolia Autonomous Region (2021GG0108), the 2022 Inner Mongolia Autonomous Region Youth Science and Technology Talent Development Plan (NJYT22044), Grassland Ecological Protection and Restoration Treatment Subsidy (RK2200000355), Basic scientific research business expenses of universities directly under Inner Mongolia Autonomous Region (BR220106 and BR221037), Innovation Platform (Talent) Program of Inner Mongolia Autonomous Region: Key Laboratory of Grassland Management and Utilization, (2022PT0003), and the Natural Science Foundation of China Inner Mongolia (2023MS03025).

## Ethics Statement

This work was performed under the auspices of the Ethics Committee of Inner Mongolia Agricultural University and Use Protocol NND2022093.

## Conflicts of Interest

The authors declare no conflicts of interest.

## Data Availability

Data supporting this publication are available on Figshare: https://doi.org/10.6084/m9.figshare.27216474.
